# The Annual American Men’s Internet Survey of Behaviors of Men Who Have Sex With Men in the United States: 2017 Key Indicators Report

**DOI:** 10.2196/16847

**Published:** 2020-04-13

**Authors:** Maria Zlotorzynska, Cera Cantu, Ramona Rai, Patrick Sullivan, Travis Sanchez

**Affiliations:** 1 Department of Epidemiology Rollins School of Public Health Emory University Atlanta, GA United States

**Keywords:** HIV, internet, men who have sex with men, sexually transmitted infections: surveillance, survey

## Abstract

The American Men’s Internet Survey (AMIS) is an annual Web-based behavioral survey of men who have sex with men (MSM) who live in the United States. This Rapid Surveillance Report describes the fifth cycle of data collection (July 2017 to November 2017: AMIS 2017). The key indicators are the same as those previously reported for past AMIS cycles (December 2013 to May 2014: AMIS 2013; November 2014 to April 2015: AMIS 2014; September 2015 to April 2016: AMIS 2015; and September 2016 to February 2017: AMIS 2016). The AMIS methodology has not substantively changed since AMIS 2016. The MSM were recruited from a variety of websites using banner advertisements and email blasts. Additionally, participants from AMIS 2016 who agreed to be recontacted for future research were emailed a link to AMIS 2017. Men were eligible to participate if they were aged ≥15 years, resided in the United States, provided a valid US zone improvement plan code, and reported ever having sex with a man or identified as gay or bisexual. The analysis was limited to those who reported having oral or anal sex with a male partner in the past 12 months. We examined demographic and recruitment characteristics using multivariable regression modeling (*P*<.05) stratified by the participants’ self-reported HIV status. The AMIS 2017 round of data collection resulted in 10,049 completed surveys from MSM representing every US state, Puerto Rico, and Guam. Participants were mainly non-Hispanic white, over the age of 40 years, living in the Southern United States and urban areas, and recruited from geospatial social networking websites. The plurality (4485/10,049, 44.6%) of participants was in the 40 years and older age group, followed by the youngest age group, 15 to 24 years (2726/10,049, 27.1%). Self-reported HIV prevalence was 9.6% (964/10,049). Compared with HIV-negative or unknown-status participants, HIV-positive participants were more likely to have had anal sex without a condom with a male partner in the past 12 months (adjusted odds ratio [aOR] 2.21, 95% CI 1.86-2.63) and more likely to have had anal sex without a condom with a serodiscordant or an unknown-status partner (aOR 3.13, 95% CI 2.71-3.62). The reported use of marijuana in the past 12 months was higher among HIV-positive participants than HIV-negative or unknown status participants (aOR 1.29, 95% CI 1.09-1.51). The reported use of methamphetamines and other illicit substances in the past 12 months was higher among HIV-positive participants than HIV-negative or unknown status participants (aOR 5.57, 95% CI 4.38-7.09 and aOR 1.93, 95% CI 1.65-2.27, respectively). Most HIV-negative or unknown status participants (7330/9085, 80.7%) reported ever taking an HIV test previously, and 60.6% (5504/9085) reported undergoing HIV testing in the past 12 months. HIV-positive participants were more likely to report testing and diagnosis of sexually transmitted infections than HIV-negative or unknown status participants (aOR 2.85, 95% CI 2.46-3.31 and aOR 2.73, 95% CI 2.29-3.26, respectively).

## Introduction

The American Men’s Internet Survey (AMIS) is an annual Web-based behavioral survey of men who have sex with men (MSM) who live in the United States. AMIS was developed to produce timely data from large-scale monitoring of behavior trends among MSM recruited on the Web. It was designed to complement the Centers for Disease Control and Prevention’s National HIV Behavioral Surveillance (NHBS) system, which collects data on MSM in major US cities every 3 years through venue-based recruitment [1]. An increasing number of MSM are meeting sexual partners through the internet and may have different patterns of sexual risk and HIV testing behaviors compared with MSM recruited through physical venues. AMIS is able to generate annual snapshots of behaviors in a large sample of internet-using MSM with broad geographic diversity as a supplement to venue-based studies, such as the NHBS system. We were also able to collect, update, and share state-level data with public health authorities to inform issues of local relevance by using AMIS.

The methods and past AMIS cycle data (AMIS 2013, AMIS 2014, AMIS 2015, and AMIS 2016) have been previously published [2-5].

This supplemental report has updated the existing information with data collected in AMIS 2017. The methods in AMIS 2017 have not changed from the previously published methods, unless otherwise noted. An in-depth analysis and discussion of multiyear trends for indicators reported herein has been published and includes data for the first 4 cycles of AMIS (AMIS 2013 to AMIS 2016) [6].

## Methods

### Recruitment and Enrollment

Similar to the previous year’s recruitment process, AMIS participants were recruited through convenience sampling from a variety of websites using banner advertisements or email blasts to members of the website (hereafter referred to generically as *ads*). For AMIS 2017, data were collected from July 2017 to November 2017. The survey was not incentivized. Data on the number of clicks on all banner ads were obtained directly from the websites. Men who clicked on the ads were taken directly to the survey website hosted on a secure server administered by SurveyGizmo (Boulder, Colorado). Recruitment was also done by emailing participants from the previous cycle of AMIS (AMIS 2016) who consented to be recontacted for future studies. To be eligible for the survey, participants had to be aged ≥15 years, be a cisgender male, reside in the United States, and report that they either had oral or anal sex with a male partner at least once in the past or identify as gay or bisexual (hereafter referred to as MSM). Persons who were aged <15 years or refused to provide their age were not asked any other screening questions. MSM who met the eligibility criteria and consented to participate in the study started the Web-based survey immediately. The full questionnaire for AMIS 2017 is presented in Multimedia Appendix 1.

Several data cleaning steps were performed on the raw dataset of eligible responses to obtain the final analysis dataset, in the same manner as in previous AMIS cycles [2-6]. Briefly, these steps were as follows: deduplication; limiting to surveys deemed successful, ie, observations with no missing values for the first question of at least two consecutive sections; limiting to participants who reported having oral or anal sex with a male partner in the past 12 months; and zone improvement plan (ZIP) code validation. These steps are further described in detail.

First, to deduplicate survey responses, demographic data for near-complete (>70%) survey responses with nonunique internet protocol addresses were compared, and responses that showed a 100% match for all characteristics were considered to be duplicate responses. Only the observation with highest survey completion was retained. The dataset was, then, limited to those surveys that were deemed successful. Finally, the dataset was restricted to include participants who reported having oral or anal sex in the past 12 months and who provided a valid US ZIP code. ZIP codes were validated in the same manner as done in AMIS 2016 [5]. Valid US ZIP codes were those that could be matched to the ZIP code of county crosswalk files created by the US Department of Housing and Urban Development [7]. Any ZIP codes that could not be matched to this list were, then, hand-validated by checking against the ZIP code locator tool in the US Postal Service website [8]. ZIP codes that could not be found were classified as invalid.

### Measures and Analyses

For the AMIS 2017 analyses, participants were categorized as either AMIS 2016 participants who took the survey again or new participants from the website/app based on the target audience and purpose: gay social networking (n=2), gay general interest (n=1), general social networking (n=4), and geospatial social networking (n=2). Recruitment outcomes and demographic characteristics for the AMIS 2017 participants are presented in Tables 1 and 2, and thereafter, they are recategorized according to their original source of recruitment. We did not provide the names of the websites/apps to preserve operator and client privacy, particularly when a category has only 1 operator. Participants whose data were eligible, unduplicated, and successful and who provided consent, reported having male-male sex in the past 12 months, and provided a valid US ZIP code were included in analyses of participant characteristics and behavior.

To facilitate comparisons, the key indicators and analytic approach used in AMIS were designed to mirror those used by the NHBS system [9]. Population density was defined in the same manner as defined in AMIS 2016 and was based on the National Center for Health Statistics Rural-Urban classification scheme for counties [10]. The self-reported HIV status was categorized as HIV-positive, and HIV-negative or unknown status, consistent with surveillance reports produced by the NHBS system [9]. In total, 3 substance use behaviors in the past 12 months were assessed: use of nonprescribed marijuana, use of methamphetamines, and use of any illicit drug other than marijuana or methamphetamines. All other indicators assessed remained unchanged from AMIS 2016 [5].

The analysis methods for AMIS 2017 did not substantively differ from those previously published but are repeated in this report for clarity. Overall, chi-square tests were used to identify whether participant characteristics differed significantly among recruitment sources. Multivariable logistic regression modeling was used to determine significant differences in behaviors based on the self-reported HIV status while controlling for race/ethnicity, age group, NHBS city residency, and type of recruitment website. The metropolitan statistical areas included in the NHBS system in 2017 were as follows: Atlanta, Georgia; Boston, Massachusetts; Chicago, Illinois; Dallas, Texas; Denver, Colorado; Detroit, Michigan; Houston, Texax; Los Angeles, California; Memphis, Tennessee; Miami, Florida; Nassau-Suffolk, New York; New Orleans, Louisiana, New York City, New York; Newark, New Jersey; Philadelphia, Philadelphia; Portland, Oregon; San Diego, California; San Francisco, California; San Juan, Puerto Rico; Seattle, Washington; Virginia Beach-Norfolk, Virginia; and Washington, District of Columbia. HIV testing behaviors were only examined among those who did not report that they were HIV positive, and these data were presented in participant characteristics. The multivariable logistic regression results were presented as Wald chi-square *P* values to denote an independently significant difference in the behavior for each subgroup compared with a reference group. Statistical significance was set at *P*<.05.

**Table 1:** Recruitment outcomes for the American Men’s Internet Survey, United States, 2017.

Recruitment outcomes			Total		Gay social networking (n=2)^a^		General gay interest (n=1)^a^		General social networking (n=4)^a^		Geospatial social networking (n=2)^a^		AMIS^b^ 2016 participants
Clicked ad, N			210,505		4700		421		191,958		13,426		N/A^c^
Screened^d^, n (%)			69,002 (32.78)		3136 (66.72)		394 (93.59)		51,472 (26.81)		12,306 (91.66)		1694
**Ineligible^e^, n (%)**			40,299 (58.40)		461 (14.70)		247 (62.69)		36,970 (71.83)		2507 (20.37)		114 (6.73)
	Not >15 years of age^f^	5297 (13.14)		34 (7.38)		2 (0.81)		5025 (13.59)		230 (9.17)		6 (5.26)	
	Not male^f^	21,409 (53.13)		345 (74.84)		59 (23.89)		19,084 (51.62)		1832 (73.08)		89 (78.07)	
	Not MSM^g^ ever or not identifying as gay/bisexual^f^	39,528 (98.09)		414 (89.80)		68 (27.53)		36,746 (99.39)		2191 (87.40)		109 (95.61)	
	Nonresident^f^	19,997 (49.62)		280 (60.74)		236 (95.55)		17,619 (47.66)		1800 (71.80)		62 (54.39)	
Eligible^e^, n (%)			28,703 (41.60)		2675 (85.30)		147 (37.31)		14,502 (28.17)		9799 (79.63)		1580 (93.27)
Consented^h^, n (%)			21,731 (75.71)		2065 (77.20)		129 (87.76)		10,483 (72.29)		7578 (77.33)		1476 (93.42)
Unduplicated^i^, n (%)			18,346 (84.42)		1874 (90.75)		120 (93.02)		8328 (79.44)		6682 (88.18)		1342 (90.92)
Success^j^, n (%)			11,159 (60.83)		1398 (74.60)		95 (79.17)		4298 (51.61)		4170 (62.41)		1198 (89.27)
MSM in the past 12 months^k^, n (%)			10,113 (90.63)		1305 (93.35)		86 (90.53)		3675 (85.50)		3953 (94.80)		1094 (91.32)
Valid ZIP^l^ code^m^, n (%)			10,049 (99.37)		1293 (99.08)		85 (98.84)		3648 (99.27)		3931 (99.44)		1092 (99.82)

^a^Refers to the number of websites or apps in this category.

^b^AMIS: American Men’s Internet Survey.

^c^N/A: not applicable.

^d^Proportion of total participants who clicked the ad, including those who started the screening questionnaire.

^e^Proportion of total participants screened. Participants who did not complete the screening questionnaire were considered ineligible.

^f^Proportion of total ineligible participants, including those who did not respond to the question.

^g^MSM: men who have sex with men.

^h^Proportion of eligible participants.

^i^Proportion of participants who consented. Deduplication removes participants who were marked as duplicates using the internet protocol address and demographic data matching.

^j^Proportion of unduplicated participants. Success removes participants who did not pass the test for survey completeness.

^k^Proportion of successes.

^l^ZIP: zone improvement plan.

^m^Proportion of men who had sex with men in the past 12 months. Valid US ZIP codes were those that could be matched to the ZIP code for county crosswalk files created by the US Department of Housing and Urban Development. Any ZIP codes that could not be matched to this list were then hand-validated by checking against the ZIP code locator tool in the US Postal Service website. ZIP codes that could not be found were classified as invalid.

**Table 2:** Characteristics of men who have sex with men in the American Men’s Internet Survey by recruitment type, United States, 2017.

Participant characteristics		Total	Gay social networking (n=2)^a^	General gay interest (n=1)^a^	General social networking (n=3)^a^	Geospatial social networking (n=2)^a^	AMIS^b^ 2016 participants	*P* value^c^	
**Race/ethnicity, n (%)**									**<.001**
	Black, non-Hispanic	654 (6.51)	93 (7.19)	1 (1.18)	255 (6.99)	230 (5.85)	75 (6.87)		
	Hispanic	1538 (15.31)	69 (5.34)	9 (10.59)	719 (19.71)	614 (15.62)	127 (11.63)		
	White, non-Hispanic	6955 (69.21)	1056 (81.67)	70 (82.35)	2371 (64.99)	2662 (67.72)	796 (72.89)		
	Other or multiple races	687 (6.84)	51 (3.94)	4 (4.71)	234 (6.41)	315 (8.01)	83 (7.60)		
**Age (years), n (%)**									**<.001**
	15-24	2726 (27.13)	28 (2.17)	6 (7.06)	1736 (47.59)	779 (19.82)	177 (16.21)		
	25-29	1246 (12.40)	43 (3.33)	11 (12.94)	288 (7.89)	696 (17.71)	208 (19.05)		
	30-39	1592 (15.84)	113 (8.74)	18 (21.18)	358 (9.81)	887 (22.56)	216 (19.78)		
	40 or older	4485 (44.63)	1109 (85.77)	50 (58.82)	1266 (34.70)	1569 (39.91)	491 (44.96)		
**Region, n (%)**									**<.001**
	Northeast	1875 (18.66)	266 (20.57)	19 (22.35)	636 (17.43)	763 (19.41)	191 (17.49)		
	Midwest	1917 (19.08)	274 (21.19)	11 (12.94)	671 (18.39)	750 (19.08)	211 (19.32)		
	South	3849 (38.30)	448 (34.65)	31 (36.47)	1504 (41.23)	1436 (36.53)	430 (39.38)		
	West	2398 (23.86)	305 (23.59)	24 (28.24)	837 (22.94)	972 (24.73)	260 (23.81)		
	US dependent areas	10 (0.10)	0 (0)	0 (0)	0 (0)	10 (0.25)	0 (0)		
**NHBS^d^ city resident, n (%)**									**.004**
	Yes	4127 (41.07)	533 (41.22)	38 (44.71)	1393 (38.19)	1655 (42.10)	508 (46.52)		
	No	5922 (58.93)	760 (58.78)	47 (55.29)	2255 (61.81)	2276 (57.90)	584 (53.48)		
**Population density^e^, n (%)**									**<.001**
	Urban	4230 (42.09)	481 (37.20)	45 (52.94)	1449 (39.72)	1708 (43.45)	547 (50.09)		
	Suburban	2181 (21.70)	351 (27.15)	13 (15.29)	811 (22.23)	793 (20.17)	213 (19.51)		
	Small/medium metropolitan	2821 (28.07)	323 (24.98)	23 (27.06)	1104 (30.26)	1101 (28.01)	270 (24.73)		
	Rural	806 (8.02)	138 (10.67)	4 (4.71)	284 (7.79)	318 (8.09)	62 (5.68)		
**Self-reported HIV status, n (%)**									**<.001**
	Positive	964 (9.59)	145 (11.21)	12 (14.12)	268 (7.35)	433 (11.02)	106 (9.71)		
	Negative	7180 (71.45)	964 (74.56)	64 (75.29)	2268 (62.17)	2954 (75.15)	930 (85.16)		
	Unknown	1905 (18.96)	184 (14.23	9 (10.59)	1112 (30.48)	544 (13.84)	56 (5.13)		
Total, n (%)		10,049 (100)	1293 (12.33)	85 (0.85)	3648 (36.30)	3931 (39.12)	1092 (10.87)	N/A^f^	

^a^Refers to the number of websites or apps in this category

^b^AMIS: American Men’s Internet Survey.

^c^A chi-square test for the difference in characteristics between recruitment types.

^d^NHBS: National HIV Behavioral Surveillance.

^e^The National Center for Health Statistics urban/rural category could not be assigned for 10 participants living in US territories.

^f^Not applicable.

## Results

### Recruitment Outcomes

AMIS 2017 was conducted from July 2017 to November 2017 and resulted in 210,505 persons clicking on the ads and landing on the study’s recruitment page (Table 1). Most persons who clicked on the ads were from general networking websites (191,958/210,505, 91.1%). Of the 3713 participants who completed the AMIS 2016 survey and were emailed links to the AMIS 2017 survey, 45.6% (1694/3713) clicked on the link. About one-third (32.8%) of all participants who landed on the study page started the screening process and 41.6% of them were eligible. The most common reason for ineligibility was not ever having male-male sex or not identifying as gay or bisexual. Three-quarter (75.7%) of participants who were eligible consented to participate in the survey. A total of 3385 (15.6%) surveys were likely from duplicate participants. Among unduplicated surveys, 60.8% were considered successful. Most successful surveys were from men who reported having sex with another male in the past 12 months (90.6%). Almost all of these surveys (10,049/10,113, 99.4%) provided a valid US ZIP code. Overall, the completion rate was 4.8%, with an analytical sample consisting of 10,049 surveys from 210,505 clicks.

### Participant Characteristics

**Figure 1:**
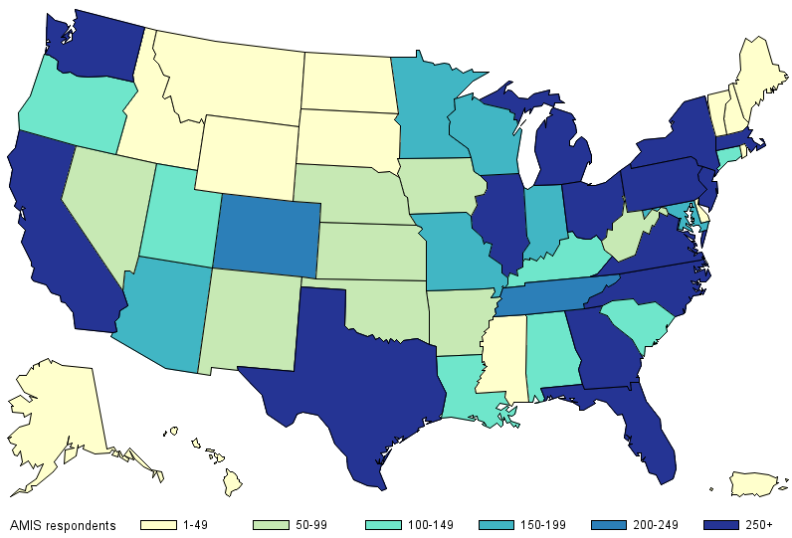
The number of men who have sex with men who participated in the American Men’s Internet Survey (AMIS) by state, 2017.

In total, 69.2% (6955/10,049) of the participants included in this report were non-Hispanic white and 44.6% were ≥40 years of age (4485/10,049); the most common region of residence was the South followed by the West (Table 2). Participants were recruited from all US states, and there were at least 100 participants each from 29 states and the District of Columbia (Figure 1). About 4 in 10 (4127/10,049, 41.1%) participants resided in an NHBS city and about the same proportion (4230/10,049, 42.1%) lived in an urban county. Overall, 9.6% (964/10,049) of participants were HIV positive, 71.5% (7180/10,049) were HIV negative, and 19.0% (1905/10,049) had an unknown HIV status. All participant characteristics differed significantly based on the recruitment source (Table 2).

### Sexual Behaviors

Around two-third (6761/10,049, 67.3%) of participants reported having anal sex without a condom with another male in the past 12 months and about one-fifth (2135/10,049, 21.3%) reported doing so with a partner of a discordant or an unknown HIV status (Table 3). Compared with HIV-negative or unknown status participants, those who were HIV positive were significantly more likely to report anal intercourse without a condom (adjusted odds ratio [aOR] 2.21, 95% CI 1.86-2.63), including with male partners who were of a discordant or an unknown status (aOR 3.13, 95% CI 2.71-3.62). Stratified by the serostatus group, anal intercourse without a condom differed significantly by race/ethnicity (HIV-negative or unknown status participants only), age group (HIV-negative or unknown status participants), and recruitment website (HIV-negative or unknown status participants only). Anal intercourse without a condom with partners of a discordant or an unknown HIV status differed significantly by age and residence in an NHBS city for HIV-negative or unknown status participants only and race/ethnicity for both HIV-negative or unknown status participants and HIV-positive status participants.

**Table 3:** Sexual behaviors with male partners of men who have sex with men in the American Men’s Internet Survey, United States, 2017.

Participant characteristics			Participants (N)	Sexual behaviors with male partners in the past 12 months				
				Anal intercourse without a condom		Anal intercourse without a condom with a partner of a discordant or an unknown HIV status		
				n (%)	*P* value^a^		n (%)	*P* value^a^
**HIV positive**			964	781 (81.01)	<.001^b^		408 (42.32)	<.001^b^
	**Race/ethnicity**							
		Black, non-Hispanic	138	102 (73.91)	.07		39 (28.26)	.01
		Hispanic	139	115 (82.73)	.54		56 (40.29)	.66
		White, non-Hispanic	617	511 (82.82)	Ref^a^		292 (47.33)	Ref^a^
		Other or multiple races	53	42 (79.25)	.70		18 (33.96)	.47
	**Age (years)**							
		15-24	39	29 (74.36)	.09		22 (56.41)	.16
		25-29	78	70 (89.74)	.05		38 (48.72)	.55
		30-39	172	151 (87.79)	.28		86 (50.00)	.75
		40 or older	675	531 (78.67)	Ref^a^		262 (38.81)	Ref^a^
	**NHBS^c^ city resident**							
		Yes	454	377 (83.04)	.11		187 (41.19)	.95
		No	510	404 (79.22)	Ref^a^		221 (43.33)	Ref^a^
	**Recruitment type**							
		Gay social networking	157	119 (75.80)	.16		80 (50.96)	.12
		General gay interest	12	11 (91.67)	.40		6 (50.00)	.80
		General social networking	332	261 (78.61)	Ref^a^		126 (37.95)	Ref^a^
		Geospatial social networking	462	389 (84.20)	.98		195 (42.21)	.34
**HIV negative or unknown status**			9085	5980 (65.82)	Ref^b^		1727 (19.01)	Ref^b^
	Race/ethnicity							
		Black, non-Hispanic	516	332 (64.34)	.34		129 (25.00)	.007
		Hispanic	1399	953 (68.12)	.008		288 (20.59)	.85
		White, non-Hispanic	6338	4186 (66.05)	Ref^a^		1161 (18.32)	Ref^a^
		Other or multiple races	634	398 (62.78)	.04		121 (19.10)	.23
	**Age (years)**							
		15-24	2687	1665 (61.97)	<.001		476 (17.72)	.02
		25-29	1168	849 (72.69)	<.001		235 (20.12)	.45
		30-39	1420	1042 (73.38)	<.001		311 (21.90)	.02
		40 or older	3810	2424 (63.62)	Ref^a^		705 (18.50)	Ref^a^
	**NHBS^c^ city resident**							
		Yes	3673	2464 (67.08)	.10		746 (20.31)	.047
		No	5412	3516 (64.97)	Ref^a^		981 (18.13)	Ref^a^
	**Recruitment type**							
		Gay social networking	1268	723 (57.02)	<.001		247 (19.48)	.42
		General gay interest	102	68 (66.67)	.98		18 (17.65)	.57
		General social networking	4022	2588 (64.35)	Ref^a^		748 (18.60)	Ref^a^
		Geospatial social networking	3688	2597 (70.42)	<.001		713 (19.33)	.74

^a^Wald chi-square *P* values from the multivariate logistic regression model comparing behavior (yes vs no) between groups with specific characteristics and a reference group (Ref).

^b^Wald chi-square *P* values from the multivariate logistic regression model comparing behavior (yes vs no) among HIV-positive participants and HIV-negative or unknown-serostatus participants. Model controlled for race/ethnicity, age, NHBS system city residency, and recruitment type.

^c^NHBS: National HIV Behavioral Surveillance.

### Substance Use Behaviors

In total, 27.6% (2775/10,049) of participants reported using marijuana, 5.9% (363/10,049) reported using methamphetamines, and 20.8% (2086/10,049) reported using other illicit substances in the past 12 months (Table 4). Compared with HIV-negative or unknown status participants, HIV-positive participants were significantly more likely to report the use of marijuana (aOR 1.29, 95% CI 1.09-1.51), methamphetamines (aOR 5.57, 95% CI 4.38-7.09), and other illicit substances (aOR 1.93, 95% CI 1.65-2.27) in the past 12 months. Among HIV-positive participants, the use of marijuana varied significantly by NHBS city residency, and the use of methamphetamines varied significantly by the recruitment website. In this group, the use of other illicit substances varied significantly by race/ethnicity and residence in an NHBS city. Additionally, the use of marijuana, methamphetamines, and other illicit substances differed significantly by age among HIV-negative or unknown status participants. In this group, the use of marijuana and other illicit substances differed significantly by race/ethnicity and residence in an NHBS city, and the use of other illicit substances differed significantly by the recruitment website.

**Table 4:** Substance use behaviors of men who have sex with men in the American Men’s Internet Survey, United States, 2017.

Participant characteristics			Participants (N)	Used marijuana			Used methamphetamines		Used other substance(s)	
				n (%)		*P* value^a^	n (%)	*P* value^a^	n (%)	*P* value^a^
**HIV positive**			964	255 (26.45)		.002^b^	136 (14.11)	<.001^b^	274 (28.42)	<.001^b^
	**Race/ethnicity**									
		Black, non-Hispanic	138	28 (20.29)		.16	10 (7.25)	.09	23 (16.67)	.003
		Hispanic	139	41 (29.50)		.24	17 (12.23)	.77	40 (28.78)	.74
		White, non-Hispanic	617	172 (27.88)		Ref^a^	100 (16.21)	Ref^a^	192 (31.12)	Ref^a^
		Other or multiple races	53	10 (18.87)		.35	5 (9.43)	.74	15 (28.30)	.58
	**Age (years)**									
		15-24	39	12 (30.77)	.76		5 (12.82)	.89	9 (23.08)	.41
		25-29	78	22 (28.21)	.95		8 (10.26)	.33	26 (33.33)	.36
		30-39	172	62 (36.05)	.20		35 (20.35)	.10	68 (39.53)	.07
		40 or older	675	159 (23.56)	Ref^a^		88 (13.04)	Ref^a^	171 (25.33)	Ref^a^
	**NHBS^c^ city resident**									
		Yes	454	135 (29.74)	.02		67 (14.76)	.27	146 (32.16)	.006
		No	510	120 (23.53)	Ref^a^		69 (13.53)	Ref^a^	128 (25.10)	Ref^a^
	**Recruitment type**									
		Gay social networking	157	31 (19.75)	.76		11 (7.01)	.02	33 (21.02)	.09
		General gay interest	12	2 (16.67)	.41		0 (0.00)	N/A^d^	5 (41.67)	.37
		General social networking	332	92 (27.71)	Ref^a^		40 (12.05)	Ref^a^	86 (25.90)	Ref^a^
		Geospatial social networking	462	129 (27.92)	.26		84 (18.18)	<.001	149 (32.25)	.66
**HIV negative or unknown status**			9085	2520 (27.74)	Ref^b^		227 (2.50)	Ref^b^	1812 (19.94)	Ref^b^
	**Race/ethnicity**									
		Black, non-Hispanic	516	132 (25.58)	.40		11 (2.13)	.27	73 (14.15)	.001
		Hispanic	1399	456 (32.59)	.33		34 (2.43)	.51	322 (23.02)	.002
		White, non-Hispanic	6338	1703 (26.87)	Ref^a^		152 (2.40)	Ref^a^	1259 (19.86)	Ref^a^
		Other or multiple races	634	166 (26.18)	.046		22 (3.47)	.08	113 (17.82)	.22
	**Age (years)**									
		15-24	2687	1016 (37.81)	<.001		35 (1.30)	.002	597 (22.22)	.09
		25-29	1168	394 (33.73)	<.001		25 (2.14)	.51	297 (25.43)	<.001
		30-39	1420	444 (31.27)	.048		55 (3.87)	.001	377 (26.55)	<.001
		40 or older	3810	666 (17.48)	Ref^a^		112 (2.94)	Ref^a^	541 (14.20)	Ref^a^
	**NHBS^c^city resident**									
		Yes	3673	1097 (29.87)	<.001		103 (2.80)	.56	823 (22.41)	<.001
		No	5412	1423 (26.29)	Ref^a^		124 (2.29)	Ref^a^	989 (18.27)	Ref^a^
	**Recruitment type**									
		Gay social networking	1268	240 (18.93)	.66		48 (3.79)	.10	190 (14.98)	.90
		General gay interest	102	25 (24.51)	.97		4 (3.92)	.88	19 (18.63)	.68
		General social networking	4022	1243 (30.91)	Ref^a^		57 (42)	Ref^a^	767 (19.07)	Ref^a^
		Geospatial social networking	3688	1012 (27.44)	.80		118 (3.20)	.46	835 (22.64)	.046

^a^Wald chi-square *P* values from the multivariate logistic regression model comparing behavior (yes vs no) between groups with specific characteristics and a reference group (Ref).

^b^Wald chi-square *P* values from the multivariate logistic regression model comparing behavior (yes vs no) among HIV-positive participants and HIV-negative or unknown-serostatus participants. Model controlled for race/ethnicity, age, National HIV Behavioral Surveillance system city residency, and recruitment type.

^c^NHBS: National HIV Behavioral Surveillance.

^d^N/A: not applicable.

### HIV Testing Behaviors

HIV testing behaviors were examined among participants who were not HIV positive (Table 5). Most participants (7330/9085, 80.7%) were previously tested for HIV infection, and 60.6% (5504/9085) were tested in the past 12 months. HIV testing behavior, both ever tested and tested in the past 12 months, differed significantly by race/ethnicity, age, residence in an NHBS city, and type of recruitment website.

**Table 5:** HIV testing behaviors of HIV-negative or unknown-status men who have sex with men in the American Men’s Internet Survey, United States, 2017.

Participant characteristics			Participants (N)		HIV testing behaviors						
					HIV tested, ever				HIV tested, past 12 months		
					n (%)		*P* value^a^		n (%)		*P* value^a^
**Race/ethnicity**											
	Black, non-Hispanic	516		445 (86.24)		.005		353 (68.41)		.01	
	Hispanic	1399		991 (70.84)		.09		814 (58.18)		.51	
	White, non-Hispanic	6338		5244 (82.74)		Ref^a^		3814 (60.18)		Ref	
	Other or multiple races	634		489 (77.13)		.23		398 (62.78)		.95	
**Age (years)**											
	15-24	2687		1478 (55.01)		<.001		1210 (45.03)		<.001	
	25-29	1168		1034 (88.53)		.05		846 (72.43)		<.001	
	30-39	1420		1310 (92.25)		<.001		1032 (72.68)		<.001	
	40 or older	3810		3508 (92.07)		Ref		2416 (63.41)		Ref	
**NHBS^b^ city resident**											
	Yes	3673		3081 (83.88)		<.001		2417 (65.80)		<.001	
	No	5412		4249 (78.51)		Ref		3087 (57.04)		Ref	
**Recruitment type**											
	Gay social networking	1268		1094 (86.28)		<.001		741 (58.44)		.03	
	General gay interest	102		94 (92.16)		.17		60 (58.82)		.24	
	General social networking	4022		2916 (72.50)		Ref		2076 (51.62)		Ref	
	Geospatial social networking	3688		3222 (87.36)		<.001		2623 (71.12)		<.001	
Total			9085		7330 (80.68)		N/A^c^		5504 (60.58)		N/A

^a^Wald chi-square *P* values from the multivariate logistic regression model comparing behavior (yes vs no) between groups with specific characteristics and a reference (Ref) group.

^b^NHBS: National HIV Behavioral Surveillance.

^c^N/A: not applicable.

### Sexually Transmitted Infection Testing and Diagnosis

In total, 42.2% (4243/10,049) of participants reported sexually transmitted infection (STI) testing in the past 12 months and just 11.5% (1153/10,049) reported a diagnosis of STI in the past 12 months. Compared with HIV-negative or unknown status participants, HIV-positive participants were significantly more likely to report STI testing (aOR 2.85, 95% CI 2.46-3.31) and STI diagnosis (aOR 2.73, 95% CI 2.29-3.26) in the past 12 months (Table 6). The most common STI diagnosis among HIV-positive participants was syphilis (137/964, 14.2%), followed by gonorrhea (116/964, 12.0%), and chlamydia (112/964, 11.6%). Chlamydia was the most common STI diagnosis among HIV-negative or unknown status participants (501/9085, 5.5%), followed by gonorrhea (481/9085, 5.3%) and syphilis (267/9085, 2.9%). STI testing significantly differed by age, residence in an NHBS city, and recruitment website among both HIV-positive status participants and HIV-negative or unknown status participants. STI testing also significantly differed by race/ethnicity for HIV-negative or unknown status participants. STI diagnosis significantly differed by race/ethnicity (HIV-negative or unknown status participants only), age (HIV-negative or unknown status participants only), residency in an NHBS city (both HIV-positive status participants and HIV-negative or unknown status participants), and recruitment website (HIV-negative or unknown status participants only).

**Table 6:** Sexually transmitted infection testing and diagnosis of men who have sex with men in the American Men’s Internet Survey, United States, 2017.

Participant characteristics			Participants (N)	STI^a^ history in the past 12 months			
				Tested for any STI		Diagnosed with any STI	
				n (%)	*P* value^b^	n (%)	*P* value^b^
**HIV positive**			964	641 (66.49)	<.001^c^	236 (24.48)	<.001^c^
	**Race/ethnicity**						
		Black, non-Hispanic	138	94 (68.12)	.82	43 (31.16)	.08
		Hispanic	139	97 (69.78)	.75	42 (30.22)	.68
		White, non-Hispanic	617	401 (64.99)	Ref^b^	133 (21.56)	Ref^b^
		Other or multiple races	53	39 (73.58)	.57	12 (22.64)	.31
	**Age (years)**						
		15-24	39	25 (64.10)	.31	13 (33.33)	.52
		25-29	78	64 (82.05)	.03	28 (35.90)	.40
		30-39	172	131 (76.16)	.44	60 (34.88)	.48
		40 or older	675	421 (62.37)	Ref^b^	135 (20.00)	Ref^b^
	**NHBS^d^ city resident**						
		Yes	454	324 (71.37)	.005	128 (28.19)	.04
		No	510	317 (62.16)	Ref^b^	108 (21.18)	Ref^b^
	**Recruitment type**						
		Gay social networking	157	96 (61.15	>.99	27 (17.20)	.29
		General gay interest	12	7 (58.33	.53	3 (25.00)	.79
		General social networking	332	193 (58.13	Ref^b^	61 (18.37)	Ref^b^
		Geospatial social networking	462	344 (74.46	.01	145 (31.39)	.07
**HIV negative or unknown status**			9085	3602 (39.65)	Ref^c^	917 (10.09)	Ref^c^
	**Race/ethnicity**						
		Black, non-Hispanic	516	248 (48.06)	.009	84 (16.28)	<.001
		Hispanic	1399	609 (43.53)	.26	177 (12.65)	.56
		White, non-Hispanic	6338	2409 (38.01)	Ref^b^	568 (8.96)	Ref^b^
		Other or multiple races	634	262 (41.32)	.11	68 (10.73)	.07
	**Age (years)**						
		15-24	2687	875 (32.56)	<.001	227 (8.45)	.004
		25-29	1168	606 (51.88)	<.001	172 (14.73)	<.001
		30-39	1420	714 (50.28)	<.001	217 (15.28)	.003
		40 or older	3810	1407 (36.93)	Ref^b^	301 (7.90)	Ref^b^
	**NHBS^d^ city resident**						
		Yes	3673	1740 (47.37)	<.001	482 (13.12)	<.001
		No	5412	1862 (34.41)	Ref^b^	435 (8.04)	Ref^b^
	**Recruitment type**						
		Gay social networking	1268	416 (32.81)	.005	74 (5.84)	.009
		General gay interest	102	42 (41.18)	.92	9 (8.82)	.92
		General social networking	4022	134 (33.37)	Ref^b^	297 (7.38)	Ref^b^
		Geospatial social networking	3688	1799 (48.78)	<.001	537 (14.56)	<.001

^a^STI: sexually transmitted infection (includes chlamydia, gonorrhea, and syphilis).

^b^Wald chi-square *P* values from the multivariate logistic regression model comparing behavior (yes vs no) between groups with specific characteristics and a reference (Ref) group.

^c^Wald chi-square *P* values from the multivariate logistic regression model comparing behavior (yes vs no) among HIV-positive participants and HIV-negative or unknown-serostatus participants. Model controlled for race/ethnicity, age, NHBS system city residency, and recruitment type.

^d^NHBS: National HIV Behavioral Surveillance.

## Appendix

Multimedia Appendix 1 American Men’s Internet Survey 2017 questionnaire.

## Abbreviations

AMIS: American Men’s Internet Survey
MSM: men who have sex with men
NHBS: National HIV Behavioral Surveillance System
STI: sexually transmitted infection
ZIP: zone improvement plan
