# Supplementary material for: The Annual American Men’s Internet Survey of Behaviors of Men Who Have Sex With Men in the United States: 2017 Key Indicators Report
Source: JMIR Public Health Surveill. 2020 Apr 13;6(2):e16847. doi: 10.2196/16847 (PMC7186873; doi:10.2196/16847)
Supplement: Multimedia Appendix 1 [file publichealth_v6i2e16847_app1.docx]

**
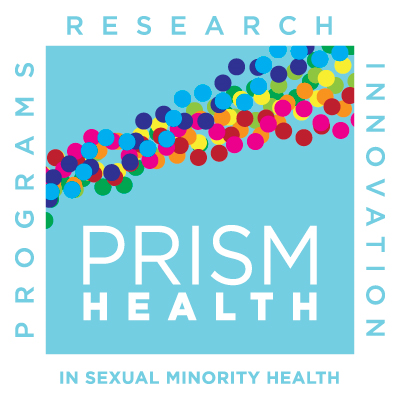
**

**American Men’s Internet Survey (AMIS) 2017:**

Online HIV Behavioral Survey of Men Who Have Sex with Men

*
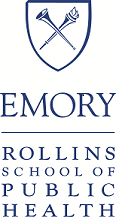
*

# Sex is the Question - 2017


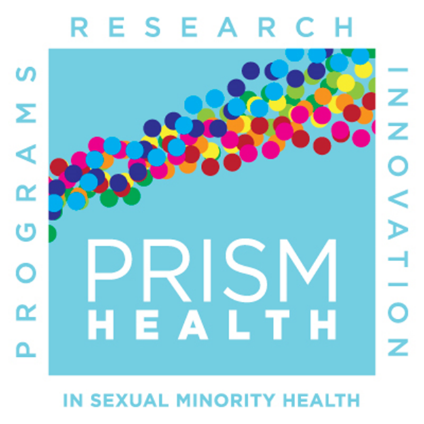


## Eligibility Screener

### Questions marked with * are required.

### How old are you?*

_________________________________________________

## Eligibility screener

#### Since July 2017, have you already completed at least part of Sex is the Question?

( ) No

( ) Yes

( ) I prefer not to answer

( ) Don't know

#### Do you consider yourself to be Hispanic or Latino?*

( ) No

( ) Yes

( ) I prefer not to answer

( ) Don't know

#### Which racial group or groups do you consider yourself to be in? Check all that apply.*

[ ] American Indian or Alaska Native

[ ] Asian

[ ] Black or African American

[ ] Native Hawaiian or Other Pacific Islander

[ ] White

[ ] I prefer not to answer

[ ] Does not apply

[ ] Don't know

#### What country do you live in?*

( ) United States

( ) Mexico

( ) Other country

### What ZIP Code do you live in?*

_________________________________________________

#### How do you describe your current gender identity? You can choose more than one answer. *

[ ] Male

[ ] Female

[ ] Transgender woman (male-to-female transgender)

[ ] Transgender man (female-to-male transgender)

[ ] Other gender identity

[ ] I prefer not to answer

[ ] Don't know

### What is your other gender identity?

_________________________________________________

#### What sex were you assigned at birth?*

( ) Male

( ) Female

( ) Intersex/ambiguous

( ) I prefer not to answer

( ) Don't know

#### Have you ****ever**** had vaginal sex (penis in the vagina) or anal sex (penis in the butt) with a woman?*

( ) No

( ) Yes

( ) I prefer not to answer

( ) Don't know

#### Have you ****ever**** had oral sex (mouth on the penis) with a man?*

( ) No

( ) Yes

( ) I prefer not to answer

( ) Don't know

#### Have you ****ever**** had anal sex (penis in the butt) with a man?*

( ) No

( ) Yes

( ) I prefer not to answer

( ) Don't know

#### Do you consider yourself to be:*

( ) Homosexual or Gay

( ) Heterosexual or Straight

( ) Bisexual

( ) I prefer not to answer

( ) Don't know

## Consent

### Thank you for your interest in our survey. The video below will give you more information about the survey. Please watch it and indicate below whether you agree to participate.*

( ) I agree to participate in the survey.

( ) I do not agree to participate in the survey.

## Assent

### Thank you for your interest in our survey! The video below will give you more information. Please watch it and indicate below whether you agree to participate.*

( ) I have read the information below. I agree to participate in this survey.

( ) I do not agree to participate in the survey.

## Demographics

#### What is the highest level of education you completed?

( ) Never attended school

( ) Less than high school

( ) Some high school

( ) High school diploma or GED

( ) Some college, Associate’s Degree, or Technical Degree

( ) College, post graduate or professional school

( ) I prefer not to answer

( ) Don't know

#### What was your household income last year from all sources before taxes? That is, the total amount of money earned ****and shared**** by all people living in your household.

( ) $0 to $19,999 annually ($0 to $1667 monthly)

( ) $20,000 to $39,999 annually ($1668 to $3333 monthly)

( ) $40,000 to $74,999 annually ($3334 to $6250 monthly)

( ) $75,000 or more annually ($6251 or more monthly)

( ) I prefer not to answer

( ) Don't know

### ****Including yourself****, how many people depend on this income?

_________________________________________________

### How many of your dependents are under the age of 18?

_________________________________________________

## Demographics: Food Security

#### ****In the past 12 months****, did you or other adults in the household ever cut the size of your meals or skip meals because there wasn't enough money for food?

( ) No

( ) Yes

( ) I prefer not to answer

( ) Don't know

#### How often did this happen?

( ) Almost every month

( ) Some months but not every month

( ) Only 1 or 2 months

( ) I prefer not to answer

( ) Don't know

## Housing

#### ****In the past 12 months,**** did you double up or stay overnight with friends, relatives, or someone you didn’t know well because you didn’t have a regular, adequate, and safe place to stay at night?

( ) No

( ) Yes

( ) I prefer not to answer

( ) Don't know

#### ****In the past 12 months****, were you ever homeless? That is, were you living on the street, in a shelter, in a Single Room Occupancy hotel (SRO), or in a car?

( ) No

( ) Yes

( ) I prefer not to answer

( ) Don't know

## Healthcare

#### What kind of health insurance or health care coverage do you currently have? Choose all that apply.

[ ] A private health plan purchased through an employer

[ ] A private health plan purchased through an exchange (i.e. Obamacare)

[ ] Medicaid or Medicare

[ ] Some other Medical Assistance program

[ ] TRICARE (CHAMPUS)

[ ] Veterans Administration coverage

[ ] Some other health care plan

[ ] I don't currently have any health insurance

[ ] I prefer not to answer

[ ] Don't know

#### ****In the past 12 months****, have you seen a doctor, nurse, or other health care provider about your own health?

( ) No

( ) Yes

( ) I prefer not to answer

( ) Don't know

#### At any of those times you were seen by a doctor or health care provider, were you offered an HIV test? An HIV test checks whether someone has the virus that causes AIDS.

( ) No

( ) Yes

( ) I prefer not to answer

( ) Don't know

#### Did your doctor or health care provider talk to you about sex (gay or straight) or sexual health?

( ) No

( ) Yes

( ) I prefer not to answer

( ) Don't know

## Outness

#### Do you consider yourself to be:

( ) Heterosexual or Straight

( ) Homosexual or Gay

( ) Bisexual

( ) Other sexual identity

( ) I prefer not to answer

( ) Don't know

### What is your sexual identity?

_________________________________________________

#### Have you ****ever**** told anyone that you are attracted to or have sex with men?

( ) No

( ) Yes

( ) I prefer not to answer

( ) Don't know

#### Who of the following people have you told that you are attracted to or have sex with men?

|  | **No** | **Yes** | **Does not apply** |
| --- | --- | --- | --- |
| Gay, lesbian, or bisexual friends | ( ) | ( ) | ( ) |
| Friends who are not gay, lesbian, or bisexual | ( ) | ( ) | ( ) |
| Family members | ( ) | ( ) | ( ) |
| Health care provider | ( ) | ( ) | ( ) |
| Employer | ( ) | ( ) | ( ) |
| Fellow employees | ( ) | ( ) | ( ) |

## Marriage

#### Have you ****ever**** been legally married?

( ) No

( ) Yes

#### Are you currently legally married?

( ) No

( ) Yes

#### What is the gender of the partner to whom you are legally married?

( ) Male

( ) Female

( ) Transgender (Male-to-Female)

( ) Transgender (Female-to-Male)

## Substance Use: Injection Drug Use

#### Have you ****ever**** in your life shot up or injected any drugs other than those prescribed for you? By shooting up, we mean anytime you might have used drugs with a needle, either by mainlining, skin popping, or muscling.

( ) No

( ) Yes

( ) I prefer not to answer

( ) Don't know

#### ****In the past 12 months****, on average, how often did you inject?

( ) More than once a day

( ) Once a day

( ) More than once a week

( ) Once a week

( ) More than once a month

( ) Once a month

( ) Less than once a month

( ) Never

( ) I prefer not to answer

( ) Don't know

#### Which drug do you inject ****most often****?

( ) Speedball - Heroin and cocaine together

( ) Heroin, by itself

( ) Cocaine, by itself

( ) Crack

( ) Crystal, meth, tina, crank, ice

( ) Something else (Specify): _______________________

( ) I prefer not to answer

( ) Don't know

## Substance Use: Non-Injection Drug Use

#### ****In the past 12 months****, have you used any non-injection drugs (drugs you did ****not**** inject), other than those prescribed for you.

( ) No

( ) Yes

( ) I prefer not to answer

( ) Don't know

#### ****In the past 12 months****, which drugs that were not prescribed to you did you use? (Check all that apply.)

[ ] Marijuana

[ ] Powdered cocaine (smoked or snorted)

[ ] Poppers (amyl nitrate)

[ ] X or Ecstasy

[ ] Painkillers (Oxycontin, Vicodin, Percocet)

[ ] Downers (Valium, Ativan, Xanax)

[ ] Crystal meth (tina, crank, ice)

[ ] Hallucinogens (LSD, mushrooms)

[ ] Special K (ketamine)

[ ] GHB

[ ] Crack cocaine

[ ] Other drug: _________________________________

[ ] Heroin (smoked or snorted)

[ ] I prefer not to answer

[ ] Don't know

## Substance Use: Drug use frequency

#### ****In the past 12 months****, how often did you use [DRUG NAME]?

( ) More than once a day

( ) Once a day

( ) More than once a week

( ) Once a week

( ) More than once a month

( ) Once a month

( ) Less than once a month

( ) I prefer not to answer

( ) Don't know

## Substance Use: Legal Marijuana

#### ****In the past 12 months****, have you been prescribed marijuana and had it filled at a legal dispensary?

( ) No

( ) Yes

( ) I prefer not to answer

( ) Don't know

## Sexual Behavior: Female Sex Partners

#### ****In the past 12 months**** (since in [MONTH/YEAR]), what types of sex have you had with a woman? (Check all that apply.)

[ ] Oral sex

[ ] Vaginal sex

[ ] Anal sex

[ ] Some other type of sex

[ ] I have not had any type of sex with a woman in the past 12 months

[ ] I prefer not to answer

[ ] Don't know

## Sexual Behavior: Female Sex Partners

#### Was the woman you had sex with that ****last time**** a main partner (someone you felt committed to above anyone else) or a casual partner (someone you didn't feel committed to or don’t know very well)?

( ) Main sex partner

( ) Casual sex partner

( ) I prefer not to answer

( ) Don't know

#### When you had sex that ****last time****, did you have either vaginal ****or**** anal sex?

( ) No

( ) Yes

( ) I prefer not to answer

( ) Don't know

#### ****The last time**** you had sex with a woman, did you have either vaginal ****or**** anal sex ****without**** ****using a condom****?

( ) No

( ) Yes

( ) I prefer not to answer

( ) Don't know

#### ****The last time**** you had sex with this partner, did you know her HIV status?

( ) No

( ) Yes

( ) I prefer not to answer

#### What was her HIV status?

( ) HIV-negative

( ) HIV-positive

( ) Indeterminate

( ) I prefer not to answer

## Sexual Behavior: Male Sex Partners

### How old were you the ****first**** time you had ****oral sex**** (mouth on the penis) with a man?

_________________________________________________

### How old were you the ****first**** time you had ****anal sex**** (penis in the butt) with a man?

_________________________________________________

#### Do you consider yourself to be a top, bottom, or versatile?

( ) Top

( ) Bottom

( ) Versatile

( ) I prefer not to answer

( ) Don't know

## Sexual Behavior: Male Sex Partners, 2

#### ****In the past 12 months**** (since in [MONTH/YEAR]), what types of sex have you had with other men?

[ ] Oral sex

[ ] Anal sex

[ ] Some other type of sex

[ ] I have not had any type of sex with a man in the past 12 months

[ ] I prefer not to answer

[ ] Don't know

### Please specify other type of sex:

_________________________________________________

### ****In the past 12 months**** (since in [MONTH/YEAR]), with how many different men have you had oral ****or**** anal sex?

_________________________________________________

### ****In the past 12 months****(since in [MONTH/YEAR]), with how many different men have you had ****anal**** sex?

_________________________________________________

### ****In the**** ****past 12 months****, since in [MONTH/YEAR], with how many different men have you had ****oral**** sex?

_________________________________________________

## Sexual Behavior: Male Sex Partners, 3

### Of the [question('value'), id='233'] men you had oral or anal sex with ****in the past 12 months****(since [MONTH/YEAR]), how many of them did you have ****anal**** sex with?

_________________________________________________

## Sexual Behavior: Condom Use

#### ****In the past 12 months****(since in [MONTH/YEAR]), did you have anal sex ****without using a condom****?

( ) No

( ) Yes

( ) I prefer not to answer

( ) Don't know

## Sexual Behavior: Condom Use, 2

### ****In the past 12 months****(since in [MONTH/YEAR]), with how many of these [NUMBER OF ANAL SEX PARTNERS] male anal sex partners did you have anal sex ****without using a condom****?

_________________________________________________

### ****In the past 12 months****(since [MONTH/YEAR]), with how many of these [NUMBER OF ANAL SEX PARTNERS] male anal sex partners did you have anal sex ****without using a condom****?

_________________________________________________

## Sexual Behavior: Male Sex Partners (1 Partner)

#### ****In the past 12 months**** (since [MONTH/YEAR]), this male partner was a:

( ) Main partner (someone you felt committed to above anyone else)

( ) Casual partner (someone you didn't feel committed to or don't know very well)

( ) I prefer not to answer

( ) Don't know

#### Did you know his HIV status?

( ) No

( ) Yes

( ) I prefer not to answer

#### What was his HIV status?

( ) HIV-negative

( ) HIV-positive

( ) Indeterminate

( ) I prefer not to answer

## Sexual Behavior: Male Sex Partners (>1)

#### ****In the past 12 months****(since [MONTH/YEAR]), the [TOTAL NUMBER SEX PARTNERS] male partners you told us about were:

( ) Only main partners (you felt committed to above anyone else)

( ) Only casual partners (you didn't feel committed to or don't know very well)

( ) Both main and casual partners

( ) I prefer not to answer

( ) Don't know

#### ****In the past 12 months**** (since [MONTH/YEAR]), did you have anal sex ****without using a condom**** with a man whose ****HIV status you did not know****?

( ) No

( ) Yes

( ) I prefer not to answer

( ) Don't know

#### Was this with a main or casual partner?

( ) Main partner

( ) Casual partner

( ) Both main and casual partners

( ) I prefer not to answer

( ) Don't know

#### ****In the past 12 months**** (since [MONTH/YEAR]) , did you have anal sex ****without using a condom**** with a man who was ****HIV positive****?

( ) No

( ) Yes

( ) I prefer not to answer

( ) Don't know

#### Was this with a main or casual partner?

( ) Main partner

( ) Casual partner

( ) Both main and casual partners

( ) I prefer not to answer

( ) Don't know

#### ****In the past 12 months****(since [MONTH/YEAR]), did you have anal sex ****without using a condom**** with a man who was ****HIV negative****?

( ) No

( ) Yes

( ) I prefer not to answer

( ) Don't know

#### Was this with a main or casual partner?

( ) Main partner

( ) Casual partner

( ) Both main and casual partners

( ) I prefer not to answer

( ) Don't know

## Sexual Behavior: Male Sex Partners (Last Sex)

### What are the initials of or a nickname for your last male sex partner?

_________________________________________________

## Sexual Behavior: Male Sex Partners (Last Sex)

### When was the ****last time**** you had either oral or anal sex with [INITIALS/NICKNAME]?

#### Month:

( ) January

( ) February

( ) March

( ) April

( ) May

( ) June

( ) July

( ) August

( ) September

( ) October

( ) November

( ) December

Year: _________________________________________________

#### Was [INITIALS/NICKNAME] a main partner (someone you felt committed to above anyone else) or a casual partner (someone you didn't feel committed to or don't know very well)?

( ) Main sex partner

( ) Casual sex partner

( ) I prefer not to answer

( ) Don't know

#### Sexual Behavior: Male Sex Partners (Last Sex)

#### That ****last time**** you had sex with [INITIALS/NICKNAME], did you have receptive anal sex where he put his penis in your anus (you were the bottom)?

( ) No

( ) Yes

( ) I prefer not to answer

( ) Don't know

#### During that ****last time**** you had receptive anal sex, did [INITIALS/NICKNAME] use a condom?

( ) No

( ) Yes, but not the whole time

( ) Yes, the whole time

( ) I prefer not to answer

( ) Don't know

#### When you had sex that ****last time****, did you have insertive anal sex where you put your penis in his anus (you were the top)?

( ) No

( ) Yes

( ) I prefer not to answer

( ) Don't know

#### During insertive anal sex that ****last time****, did you use a condom?

( ) No

( ) Yes, but not the whole time

( ) Yes, the whole time

( ) I prefer not to answer

( ) Don't know

## Sexual Behavior: Male Sex Partners (Last sex HIV)

#### The ****last time**** you had sex with [INITIALS/NICKNAME], did you know his HIV status?

( ) No

( ) Yes

( ) I prefer not to answer

#### What was [INITIALS/NICKNAME]'s HIV status?

( ) HIV-negative

( ) HIV-positive

( ) Indeterminate

( ) I prefer not to answer

## Last sex partner - age

#### What was [INITIALS/NICKNAME]'s age?

( ) 19 years or younger

( ) 20 to 24 years

( ) 25 to 29 years

( ) 30 to 34 years

( ) 35 to 39 years

( ) 40 to 44 years

( ) 45 to 49 years

( ) 50 to 54 years

( ) 55 to 59 years

( ) 60 to 64 years

( ) 65 to 69 years

( ) 70 to 74 years

( ) 75 to 79 years

( ) 80 years or older

( ) Don't know

( ) Prefer not to answer

#### Was [INITIALS/NICKNAME] younger than you, older than you, or the same age as you?

[ ] Younger

[ ] Older

[ ] Same age

[ ] I prefer not to answer

[ ] Don't know

#### Which of the following best describes [INITIALS/NICKNAME]'s ethnic background?

[ ] American Indian or Alaska Native

[ ] Asian

[ ] Black or African American

[ ] Hispanic or Latino

[ ] Native Hawaiian or other Pacific Islander

[ ] White

[ ] I prefer not to answer

[ ] Don't know

#### Did you have sex with [INITIALS/NICKNAME] one time ('one night stand'), or more than one time?

( ) One time

( ) More than one time

( ) I prefer not to answer

( ) Don't know

### How long have you been having a sexual relationship with [INITIALS/NICKNAME]?

_________________________________________________

( ) Days

( ) Months

( ) Years

#### Do you expect to have sex with [INITIALS/NICKNAME] again?

( ) No

( ) Yes

( ) I prefer not to answer

( ) Don't know

## Sexual Behavior: Male Sex Partners (Last sex relationship)

#### As far as you know, during the time you were having a sexual relationship with [INITIALS/NICKNAME], did ****he**** have sex with other people? Would you say he:

( ) Definitely did not

( ) Probably did not

( ) Probably did

( ) Definitely did

( ) I prefer not to answer

( ) Don't know

#### During the time you were having a sexual relationship with [INITIALS/NICKNAME], did ****you**** have sex with other people?

( ) No

( ) Yes

( ) I prefer not to answer

( ) Don't know

#### Sexual Behavior: Male Sex Partners (Last sex relationship)

#### Where did you ****first**** meet [INITIALS/NICKNAME]?

( ) Work

( ) School

( ) House party

( ) Mobile phone app (such as a gay chat, dating or hookup app)

( ) Internet

( ) Bar/Club

( ) Circuit party or rave

( ) Public sex environment (such as a bathhouse, sex club, sex resort, cruising area, private sex party, or adult bookstore)

( ) Place or worship (such as a church, synagogue, mosque)

( ) Other: _________________________________________________

( ) I prefer not to answer

( ) Don't know

## Alcohol and Drugs at last sex

#### Before or during the ****last time**** you had sex with [INITIALS/NICKNAME], did you use:

( ) Alcohol

( ) Drugs

( ) Both alcohol and drugs

( ) Neither one

( ) I prefer not to answer

( ) Don't know

## Alcohol and Drugs at last sex

### Before or during sex the ****last time**** you had sex with [INITIALS/NICKNAME], how many alcoholic drinks did you have?

_________________________________________________

## Alcohol and Drugs at last sex

#### The ****last time**** you had sex with [INITIALS/NICKNAME], which drugs did you use? Check all that apply.

[ ] Other drug: ___________________________

[ ] I prefer not to answer

[ ] Don't know

## Sexual Behavior: Social Habits

#### ****In the**** ****past 12 months****, have you exchanged things like money or drugs for sex with a male partner? Check all that apply.

[ ] No

[ ] Yes, I gave a sex partner things like drugs or money for sex

[ ] Yes, a sex partner gave me things like drugs or money for sex

[ ] I prefer not to answer

[ ] Don't know

#### ****In the past 12 months****, how often have you gone to a place (not online) where gay men hangout, meet or socialize? These could include bars, clubs, social organizations, parks, gay businesses, bookstores, sex clubs, etc.

( ) More than once a day

( ) Once a day

( ) More than once a week

( ) Once a week

( ) More than once a month

( ) Once a month

( ) Less than once a month

( ) Never

( ) I prefer not to answer

( ) Don't know

#### ****In the**** ****past 12 months****, have you used any of following kinds of internet sites to ****meet or socialize with gay men?****

[ ] Social network websites (such as Facebook)

[ ] Dating websites directed towards gay men

[ ] Mobile phone apps (such as gay chat, dating and hookup apps)

[ ] None of the above

[ ] I prefer not to answer

[ ] Don't know

#### ****In the**** ****past 12 months****, how often did you use social network websites (such as Facebook) ****to meet or socialize with gay men?****

( ) More than once a day

( ) Once a day

( ) More than once a week

( ) Once a week

( ) More than once a month

( ) Once a month

( ) Less than once a month

( ) Never

( ) I prefer not to answer

( ) Don't know

#### ****In the**** ****past 12 months****, how often have you used dating websites directed towards gay men to ****meet or socialize with gay men?****

( ) More than once a day

( ) Once a day

( ) More than once a week

( ) Once a week

( ) More than once a month

( ) Once a month

( ) Less than once a month

( ) Never

( ) I prefer not to answer

( ) Don't know

#### ****In the**** ****past 12 months****, how often did you use mobile phone apps (such as gay chat, dating and hookup apps) ****to meet or socialize with gay men?****

( ) More than once a day

( ) Once a day

( ) More than once a week

( ) Once a week

( ) More than once a month

( ) Once a month

( ) Less than once a month

( ) Never

( ) I prefer not to answer

( ) Don't know

#### How satisfied are you with your current sex life?

( ) Very satisfied

( ) Satisfied

( ) Unsure

( ) Dissatisfied

( ) Very Dissatisfied

( ) I prefer not to answer

( ) Don't know

## Chicago DPH questions

#### ****In the past 12 months****, when you met someone through the internet or mobile apps, how frequently did that lead to sex?

( ) Every time I met someone

( ) Sometimes when I met someone

( ) Rarely when I met someone

( ) Never when I met someone

( ) I prefer not to answer

( ) Don't know

#### Did you discuss HIV status with individuals you met through the internet or mobile apps prior to sex?

( ) No

( ) Sometimes

( ) Yes

( ) I prefer not to answer

( ) Don't know

## HIV Testing

#### Have you ****ever**** been tested for HIV? An HIV test checks whether someone has the virus that causes AIDS.

( ) No

( ) Yes

( ) I prefer not to answer

( ) Don't know

### ****In the past 2 years**** (since [system("date"),format="F"] of 2015), how many times have you been tested for HIV?

_________________________________________________

### When did you have your ****most recent**** HIV test? If you don't know the exact month, please enter your best guess.

#### Month:

( ) January

( ) February

( ) March

( ) April

( ) May

( ) June

( ) July

( ) August

( ) September

( ) October

( ) November

( ) December

Year: _________________________________________________

## HIV Testing

#### Have you had an HIV test ****in the past 12 months**** (since in [MONTH/YEAR])?

( ) No

( ) Yes

( ) I prefer not to answer

( ) Don't know

#### When you most recently got tested in [MONTH/YEAR], where did you get tested?

( ) Private doctor's office

( ) HIV counseling and testing site

( ) Public health clinic/community health clinic

( ) Street outreach program/mobile unit

( ) Sexually transmitted disease clinic

( ) Hospital (inpatient)

( ) Correctional facility (jail or prison)

( ) Emergency room

( ) At home

( ) Other

( ) I prefer not to answer

( ) Don't know

## HIV Status

#### What was the result of your ****most recent**** HIV test in [MONTH/YEAR]?

( ) Negative

( ) Positive

( ) Never obtained results

( ) Indeterminate

( ) I prefer not to answer

( ) Don't know

#### Before your most recent test in [MONTH/YEAR], did you ****ever**** test positive for HIV?

( ) No

( ) Yes

( ) I prefer not to answer

( ) Don't know

#### Was your most recent test in [MONTH/YEAR] your ****first**** positive test?

( ) No

( ) Yes

( ) I prefer not to answer

( ) Don't know

### When did you ****first**** test positive?

#### Month

( ) January

( ) February

( ) March

( ) April

( ) May

( ) June

( ) July

( ) August

( ) September

( ) October

( ) November

( ) December

Year: _________________________________________________

## HIV Positive

#### Are you currently taking antiretroviral medicines to treat your HIV infection?

( ) No

( ) Yes

( ) I prefer not to answer

( ) Don't know

#### What is the ****main**** reason you are not currently taking any antiretroviral medicines?

( ) Not currently going to a health care provider for my HIV

( ) CD4 count and viral load are good

( ) Don't have money or insurance for antiretroviral medicines

( ) Don't want to take antiretroviral medicines

( ) Other

( ) I prefer not to answer

( ) Don't know

## HIV Concern - Chicago DPH

#### How likely do you think you are at risk for acquiring HIV?

( ) Not at all

( ) Somewhat likely

( ) Likely

( ) Very likely

( ) I prefer not to answer

( ) Don’t know

## PrEP Module

#### ****Before today****, have you ****ever**** heard of people who do not have HIV taking PrEP, the antiretroviral medicine taken every day for months or years to reduce the risk of getting HIV?

( ) No

( ) Yes

#### ****In the past 12 months****(since in [MONTH/YEAR]), have you had a discussion with a health care provider about taking PrEP?

( ) No

( ) Yes

( ) I prefer not to answer

( ) Don't know

#### ****In the past 12 months****(since in [MONTH/YEAR]), have you taken PrEP?

( ) No

( ) Yes

( ) I prefer not to answer

( ) Don't know

#### Which of the following people or places describe how you got PrEP during the last 12 months? Check all that apply.

[ ] Prescription from a healthcare provider

[ ] Directly from a healthcare provider or clinic

[ ] A friend or relative

[ ] A person you had/have sex with

[ ] Some other source

[ ] I prefer not to answer

[ ] Don't know

### What was that other source for PrEP?

_________________________________________________

## Current PrEP Use

#### Are you currently taking PrEP?

( ) No

( ) Yes

( ) I prefer not to answer

( ) Don't know

#### ****In the last 30 days****, about how many doses of PrEP did you take?

( ) Less than 15

( ) 16-29

( ) 30

#### The ****last time**** you were on PrEP, how many months in a row were you taking it?

( ) Less than 2 months

( ) 2 to 6 months

( ) 7 to 12 months

( ) I prefer not to answer

( ) Don't know

#### How many months in a row have you been taking PrEP?

( ) Less than 2 months

( ) 2 to 6 months

( ) 7 to 12 months

( ) I prefer not to answer

( ) Don't know

## PrEP, continued

#### Would you be willing to take anti-HIV medicines ****every day**** to lower your chances of getting HIV?

( ) No

( ) Yes

( ) I prefer not to answer

( ) Don't know

#### If you were to start taking PrEP, where would you go to access it?

( ) STI clinic

( ) Primary care physician

( ) Emergency room

( ) Unsure

( ) Other – please describe: __________________________

## CDC PrEP After Sex

#### Researchers are also working on a form of PrEP that does not require you taking a daily pill. Instead, you would take only one pill ****within 2 hours after**** you had sex. How likely would you be to use this type of PrEP that is taken within 2 hours after you had sex?

( ) Very likely

( ) Somewhat likely

( ) Neither likely or unlikely

( ) Somewhat unlikely

( ) Very unlikely

#### Researchers are also working on a form of PrEP that does not require you taking a daily pill. Instead, you would take only one pill ****within 12-24 hours after**** you had sex. How likely would you be to use this type of PrEP that is taken within 12-24 hours after you had sex?

( ) Very likely

( ) Somewhat likely

( ) Neither likely or unlikely

( ) Somewhat unlikely

( ) Very unlikely

## STD Concern

#### How concerned are you about becoming infected with a sexually transmitted disease other than HIV (i.e., syphilis, chlamydia, gonorrhea)?

( ) Very concerned

( ) Somewhat concerned

( ) Neither concerned nor unconcerned

( ) Somewhat unconcerned

( ) Not concerned at all

( ) Don't know

## STD-PrEP

#### How likely would you be to use "STD-PrEP" as a ****daily pill**** were it available?

( ) Very likely

( ) Somewhat likely

( ) Neither likely nor unlikely

( ) Somewhat unlikely

( ) Very unlikely

( ) Don't know

#### How likely would you be to use "STD-PrEP" as a ****pill that would be taken within 24 hours before sex****?

( ) Very likely

( ) Somewhat likely

( ) Neither likely nor unlikely

( ) Somewhat unlikely

( ) Very unlikely

( ) Don't know

## PEP

#### ****In the past 12 months****(since [MONTH/YEAR]), have you taken PEP to reduce the risk of getting HIV?

( ) No

( ) Yes

( ) Don't know

( ) I prefer not to answer

## STD-PEP

#### How likely would you be to use "STD-PEP" as a pill were it available?

( ) Very likely

( ) Somewhat likely

( ) Neither likely nor unlikely

( ) Somewhat unlikely

( ) Very unlikely

( ) Don't know

## Serosorting and prevention strategies

#### Which of the following describes what you did in the ****past 12 months**** to reduce your risk of getting HIV?  Check all that apply.

| [ ] I took PrEP (pre-exposure prophylaxis or Truvada) |
| --- |
| [ ] I did not have sex with HIV-positive men or men whose status I didn’t know |
| [ ] I only had oral sex or jacked off with my partner if he was HIV-positive or I didn’t know his status |
| [ ] I used condoms for anal sex with all of my male partners |
| [ ] I used condoms for anal sex if my partner was HIV-positive or I didn’t know his status |
| [ ] I topped all my male partners |
| [ ] I topped my partner if he was HIV-positive or I didn’t know his status |
| [ ] I only had anal sex without a condom with an HIV-positive partner if he was taking HIV medicines and/or he had an undetectable viral load |
| [ ] I only had anal sex without a condom with an HIV-negative partner if he was taking PrEP |
| [ ] Other - please specify: ______________________ |
| [ ] None of these |

#### Which of the following describes what you did in the ****past 12 months**** to reduce the risk of transmitting HIV?  Check all that apply.

#### [ ] I take HIV medications as prescribed

#### [ ] I did not have sex with HIV-negative men or men whose status I didn’t know

#### [ ] I only had oral sex or jacked off with my partner if he was HIV-negative or I didn’t know his status

#### [ ] I used condoms for anal sex with all of my male partners

#### [ ] I used condoms for anal sex if my partner was HIV-negative or I didn’t know his status

#### [ ] I was a bottom with all my male partners

#### [ ] I was a bottom with my partner if he was HIV-negative or I didn’t know his status

#### [ ] I only had anal sex without a condom with a partner who was HIV-negative or whose status I did not know if I had an undetectable viral load

#### [ ] I only had anal sex without a condom with an HIV-negative partner if he was taking PrEP

#### [ ] Other - please specify: ______________________

#### [ ] None of these

## Stigma - NHBS Measure

#### ****In the past 12 months****, have any of the following things happened to you because someone knew or assumed you were attracted to men?

|  | **Yes** | **No** | **I prefer not to answer** | **Don't know** | **Does not apply** |
| --- | --- | --- | --- | --- | --- |
| You were called names or insulted | ( ) | ( ) | ( ) | ( ) | ( ) |
| You received poorer services than other people in restaurants, stores, other businesses or agencies | ( ) | ( ) | ( ) | ( ) | ( ) |
| You were treated unfairly at work or school | ( ) | ( ) | ( ) | ( ) | ( ) |
| You were denied or given lower quality health care | ( ) | ( ) | ( ) | ( ) | ( ) |
| You were physically attacked or injured | ( ) | ( ) | ( ) | ( ) | ( ) |

#### How much do you agree or disagree with the following: Most people in my area are tolerant of gays and bisexuals.

( ) Strongly agree

( ) Agree

( ) Neither agree or disagree

( ) Disagree

( ) Strongly disagree

( ) I prefer not to answer

( ) Don't know

## Stigma

#### Have you ever felt excluded from family activities because you have sex with men?

( ) No

( ) Yes, in the last 6 months

( ) Yes, but not in the last 6 months

( ) I prefer not to answer

( ) Don’t know

#### Have you ever felt that family members have made discriminatory remarks or gossiped about you because you have sex with men?

( ) No

( ) Yes, in the last 6 months

( ) Yes, but not in the last 6 months

( ) I prefer not to answer

( ) Don’t know

#### Have you ever felt rejected by your friends because you have sex with men?

( ) No

( ) Yes, in the last 6 months

( ) Yes, but not in the last 6 months

( ) I prefer not to answer

( ) Don’t know

## Stigma

#### Have you ever felt afraid to go to health care services because you worry someone may learn you have sex with men?

( ) No

( ) Yes, in the last 6 months

( ) Yes, but not in the last 6 months

( ) I prefer not to answer

( ) Don’t know

#### Have you ever avoided going to health care services because you worry someone may learn you have sex with men?

( ) No

( ) Yes, in the last 6 months

( ) Yes, but not in the last 6 months

( ) I prefer not to answer

( ) Don’t know

#### Have you ever heard health care providers gossiping about you (talking about you) because you have sex with men?

( ) No

( ) Yes, in the last 6 months

( ) Yes, but not in the last 6 months

( ) I prefer not to answer

( ) Don’t know

#### Have you ever felt that you were not treated well in a health center because someone knew that you have sex with men?

( ) No

( ) Yes, in the last 6 months

( ) Yes, but not in the last 6 months

( ) I prefer not to answer

( ) Don’t know

## Stigma

#### Have you ever felt that the police refused to protect you because you have sex with men?

( ) No

( ) Yes, in the last 6 months

( ) Yes, but not in the last 6 months

( ) I prefer not to answer

( ) Don’t know

#### Have you ever felt scared to be in public places because you have sex with men?

( ) No

( ) Yes, in the last 6 months

( ) Yes, but not in the last 6 months

( ) I prefer not to answer

( ) Don’t know

#### Have you ever been verbally harassed and felt it was because you have sex with men?

( ) No

( ) Yes, in the last 6 months

( ) Yes, but not in the last 6 months

( ) I prefer not to answer

( ) Don’t know

#### Have you ever been blackmailed by someone because you have sex with men?

( ) No

( ) Yes, in the last 6 months

( ) Yes, but not in the last 6 months

( ) I prefer not to answer

( ) Don’t know

## Stigma

#### Has someone ever physically hurt you (pushed, shoved, slapped, hit, kicked, choked or otherwise physically hurt you)?

( ) No

( ) Yes, in the last 6 months

( ) Yes, but not in the last 6 months

( ) I prefer not to answer

( ) Don’t know

#### Do you believe any of these experiences of physical violence was/were related to the fact that you have sex with men?

( ) No

( ) Yes, in the last 6 months

( ) Yes, but not in the last 6 months

( ) I prefer not to answer

( ) Don’t know

#### Have you ever been forced to have sex when you did not want to? By forced, we mean physically forced, coerced to have sex, or penetrated with an object, when you did not want to.

( ) No

( ) Yes, in the last 6 months

( ) Yes, but not in the last 6 months

( ) I prefer not to answer

( ) Don’t know

#### Do you believe any of these experiences of sexual violence were related to the fact that you have sex with men?

( ) No

( ) Yes, in the last 6 months

( ) Yes, but not in the last 6 months

( ) I prefer not to answer

( ) Don’t know

## K-6 Mental Health Scale

#### During the past ****30 days****, about how often did you feel...

|  | **All of the time** | **Most of the time** | **Some of the time** | **A little of the time** | **None of the time** | **I prefer not to answer** | **Don't know** |
| --- | --- | --- | --- | --- | --- | --- | --- |
| Nervous | ( ) | ( ) | ( ) | ( ) | ( ) | ( ) | ( ) |
| Hopeless | ( ) | ( ) | ( ) | ( ) | ( ) | ( ) | ( ) |
| Restless or fidgety | ( ) | ( ) | ( ) | ( ) | ( ) | ( ) | ( ) |
| So depressed that nothing could cheer you up | ( ) | ( ) | ( ) | ( ) | ( ) | ( ) | ( ) |
| That everything was an effort | ( ) | ( ) | ( ) | ( ) | ( ) | ( ) | ( ) |
| Worthless | ( ) | ( ) | ( ) | ( ) | ( ) | ( ) | ( ) |

## Suicidality

#### At any time in the past 12 months, up to and including today, did you seriously think about trying to kill yourself?

( ) No

( ) Yes

( ) I prefer not to answer

( ) Don't know

#### During the past 12 months, did you make any plans to kill yourself?

( ) No

( ) Yes

( ) I prefer not to answer

( ) Don't know

#### During the past 12 months, did you try to kill yourself?

( ) No

( ) Yes

( ) I prefer not to answer

( ) Don't know

## Viral STI Diagnoses

#### Has a doctor, nurse or other health care provider ****ever**** told you that you had any of the following? Check all that apply.

[ ] Hepatitis

[ ] Genital herpes

[ ] Genital warts

[ ] Human papillomavirus or HPV

[ ] None of the above

[ ] I prefer not to answer

[ ] Don't know

#### What type or types of hepatitis have you had? Check all that apply.

[ ] Hepatitis A

[ ] Hepatitis B

[ ] Hepatitis C

[ ] Other

[ ] I prefer not to answer

[ ] Don't know

#### There are vaccines or shots that can prevent some types of hepatitis. Have you ****ever**** had a hepatitis vaccine?

( ) No

( ) Yes

( ) I prefer not to answer

( ) Don't know

#### What type or types of hepatitis vaccine have you had?

( ) Hepatitis A vaccine

( ) Hepatitis B vaccine

( ) Both Hepatitis A and B vaccine

( ) I prefer not to answer

( ) Don't know

## LGV - Chicago DPH

#### In the ****past 12 months****, that is, since in [MONTH/YEAR], has a health care professional discussed the risk of acquiring Lymphogranuloma Venereum (LGV) with you?

( ) No

( ) Yes

( ) I prefer not to answer

( ) Don't know

## STD Testing - New for 2017

**Have you ever been tested for the sexually transmitted infections gonorrhea, chlamydia, or syphilis?**

( ) No

( ) Yes

( ) I prefer not to answer

( ) Don't know

**In the past 12 months, that is, since in [MONTH/YEAR], were you tested by a doctor or other health care provider for a sexually transmitted infection like gonorrhea, chlamydia, or syphilis?**

( ) No

( ) Yes

( ) I prefer not to answer

( ) Don't know

**In the past 12 months, when you were tested by a doctor or other health care provider for a sexually transmitted infection like gonorrhea, chlamydia, or syphilis, what samples did you provide for testing? Check all that apply.**

[ ] I had my blood drawn

[ ] I gave a urine sample

[ ] I had my rectum (butt) swabbed

[ ] I had my throat swabbed

[ ] I prefer not to answer

[ ] Don't know

## Bacterial STI Diagnoses

#### ****In the past 12 months**** (since in [MONTH/YEAR]), has a doctor, nurse or other health care provider told you that you had any of the following? Check all that apply.

[ ] Gonorrhea

[ ] Chlamydia

[ ] Syphilis

[ ] None of the above

[ ] I prefer not to answer

[ ] Don't know

## CDC GC-CT Self-testing

### Researchers are developing self-tests for gonorrhea and chlamydia that would allow people to test themselves with self-collected swabs or urine and read the results by themselves, much like women can read the results of a pregnancy test. If such a test were available to you, where would you prefer to take the test?

( ) Home

( ) Doctor’s office

( ) Family Planning Clinic

( ) Pharmacy Clinic

( ) STD Clinic

#### If such a test became available, how much would you be willing to pay for this test?

( ) $50

( ) $40

( ) $30

( ) $20

( ) $10

( ) I wouldn’t be willing to pay anything

## HPV

#### A vaccine to prevent human papillomavirus (HPV) infection is available and is called the HPV shot, cervical cancer vaccine, GARDASIL®, or CERVARIX®. Have you ****ever**** received the HPV vaccine?

( ) No

( ) Yes

( ) I prefer not to answer

( ) Don't know

### How old were you when you received your ****first**** dose of the HPV vaccine?

_________________________________________________

## Assessment of Prevention Activities

#### ****In the**** ****past 12 months****, have you gotten any free condoms, not counting those given to you by a friend, relative, or sex partner?

( ) No

( ) Yes

( ) I prefer not to answer

( ) Don't know

#### ****In the past 12 months****, have you had a one-on-one conversation with an outreach worker, counselor, or prevention program worker about ways to prevent HIV? Don't count the times where you had a conversation as part of an HIV test.

( ) No

( ) Yes

( ) I prefer not to answer

( ) Don't know

#### ****In the**** ****past 12 months****, have you been a participant in any organized session(s) involving a small group of people to discuss ways to prevent HIV? Don't include discussions you had with a group of friends.

( ) No

( ) Yes

( ) I prefer not to know

( ) Don't know

## BHOC Questions

#### In the past 12 months which dating/hookup smartphone apps have you used? Check all that apply.

[ ] Grindr

[ ] Scruff

[ ] Tinder

[ ] Jack’d

[ ] Adam 4 Adam/RADAR

[ ] GROWLr

[ ] Mr. X

[ ] Daddyhunt

[ ] Hornet

[ ] GuySpy

[ ] Squirt

[ ] Recon

[ ] Other (please specify): ______

[ ] None of these

## BHOC Questions

#### How much would you like your favorite dating/hookup app to provide the following features? Please select a response for each feature.

|  | **I would like the app to add this** | **I am neutral on whether the app adds this** | **I would not like the app to add this** |
| --- | --- | --- | --- |
| Links to nearby HIV/STD testing centers | ( ) | ( ) | ( ) |
| Links to local PrEP providers | ( ) | ( ) | ( ) |
| HIV/STD testing reminders at an interval you choose | ( ) | ( ) | ( ) |
| A way to tell your sex partners if you get diagnosed with HIV or an STD | ( ) | ( ) | ( ) |
| Daily reminders to take HIV medications for prevention or treatment (PrEP or antiretrovirals) | ( ) | ( ) | ( ) |
| Live chat function to speak with a sexual health counselor | ( ) | ( ) | ( ) |
| A way to find free condoms near you | ( ) | ( ) | ( ) |
| A way to order free condoms to be mailed to you | ( ) | ( ) | ( ) |
| A way to order a free home HIV test to be mailed to you | ( ) | ( ) | ( ) |
| A way to keep track of or take notes about your partners on the app so you can find them again | ( ) | ( ) | ( ) |
| A way to confirm that a user was recently tested for HIV (for example, a link to a test result). | ( ) | ( ) | ( ) |
| Alerts about disease outbreaks in your area | ( ) | ( ) | ( ) |

## BHOC Questions

#### If a dating/hookup app provided any of the following features, ****how would it change what you think about the app****? Please select a response for each feature.

|  | **I would think better of the app** | **It wouldn’t change what I think of the app** | **I would think worse of the app** |
| --- | --- | --- | --- |
| Links to nearby HIV/STD testing centers | ( ) | ( ) | ( ) |
| Links to local PrEP providers | ( ) | ( ) | ( ) |
| HIV/STD testing reminders at an interval you choose | ( ) | ( ) | ( ) |
| A way to tell your sex partners if you get diagnosed with HIV or an STD | ( ) | ( ) | ( ) |
| Daily reminders to take HIV medications for prevention or treatment (PrEP or antiretrovirals) | ( ) | ( ) | ( ) |
| Live chat function to speak with a sexual health counselor | ( ) | ( ) | ( ) |
| A way to find free condoms near you | ( ) | ( ) | ( ) |
| A way to order free condoms to be mailed to you | ( ) | ( ) | ( ) |
| A way to order a free home HIV test to be mailed to you | ( ) | ( ) | ( ) |
| A way to keep track of or take notes about your partners on the app so you can find them again | ( ) | ( ) | ( ) |
| A way to confirm that a user was recently tested for HIV (for example, a link to a test result). | ( ) | ( ) | ( ) |
| Alerts about disease outbreaks in your area | ( ) | ( ) | ( ) |

## BHOC Questions - Random group 1

#### On a scale from 1-5 (1 being very poor and 5 being excellent), how would you rate your overall perception of dating/hookup apps?

( ) Very poor

( ) Poor

( ) Neutral

( ) Good

( ) Excellent

## BHOC Random group 1 - HIV positive

#### How likely would you be to review, and if necessary, update your profile choices regarding sexual health (such as using condoms, being undetectable, or STD testing) if you received a periodic message from your favorite app to do so?

( ) Very unlikely

( ) Somewhat unlikely

( ) Somewhat likely

( ) Very likely

#### If your favorite dating/hookup app offered to send you reminders to get tested for STDs ****every 3 months****, how likely would you be to accept the reminders?

( ) Very unlikely

( ) Somewhat unlikely

( ) Somewhat likely

( ) Very likely

#### If your favorite dating/hookup app offered to send you reminders to get tested for STDs ****at an interval that you choose****, how likely would you be to accept the reminders?

( ) Very unlikely

( ) Somewhat unlikely

( ) Somewhat likely

( ) Very likely

## BHOC Random group 1 - HIV negative/unknown

#### How likely would you be to review, and if necessary, update your profile choices regarding sexual health (such as using condoms, PrEP, being HIV-negative, or STD testing) if you received a periodic message from your favorite app to do so?

( ) Very unlikely

( ) Somewhat unlikely

( ) Somewhat likely

( ) Very likely

#### If your favorite dating/hookup app offered to send you reminders to get tested for HIV and STDs ****every 3 months****, how likely would you be to accept the reminders?

( ) Very unlikely

( ) Somewhat unlikely

( ) Somewhat likely

( ) Very likely

#### If your favorite dating/hookup app offered to send you reminders to get tested for HIV and STDs ****at an interval that you choose****, how likely would you be to accept the reminders?

( ) Very unlikely

( ) Somewhat unlikely

( ) Somewhat likely

( ) Very likely

## BHOC Random questions 2

#### In the past 12 months, have you used the HIV status or his preferred sexual health strategy (e.g. condoms, PrEP, staying undetectable) on a guy’s app profile when deciding whether to chat with him?

( ) No

( ) Yes

( ) I prefer not to answer

( ) Don't know

#### With your last new sex partner that you met on an app, how did you find out about his HIV status? Check all that apply.

[ ] His app profile

[ ] I talked to him about it

[ ] I didn’t know his HIV status

[ ] Other (please specify): __________

[ ] I haven’t met a sex partner on an app

#### With your last new sex partner that you met on an app, how did you find out his preferred sexual health strategy (e.g. condoms, PrEP, treatment as prevention)? Check all that apply.

[ ] His app profile

[ ] I talked to him about it

[ ] I didn’t know

[ ] Other (please specify): ___________

[ ] I haven’t met a sex partner on an app

## Prevention Campaigns

### Thank you for staying with us! You are almost done the survey.

### ****In the past 12 months****, how often did you see or hear the following slogans or messages?

#### Let's stop HIV together:

( ) Never

( ) Rarely

( ) Sometimes

( ) Often

( ) Very Often

( ) I prefer not to answer

( ) Don't know

#### On a scale of 0 to 5, where 0 means "not very effective" and 5 means "very effective", how effective do you think this slogan or message is?

( ) 0 (Not Effective)

( ) 1

( ) 2

( ) 3

( ) 4

( ) 5 (Very Effective)

#### Protest:

( ) Never

( ) Rarely

( ) Sometimes

( ) Often

( ) Very Often

( ) I prefer not to answer

( ) Don't know

#### On a scale of 0 to 5, where 0 means "not very effective" and 5 means "very effective", how effective do you think this slogan or message is?

( ) 0 (Not Effective)

( ) 1

( ) 2

( ) 3

( ) 4

( ) 5 (Very Effective)

#### ACT Against AIDS:

( ) Never

( ) Rarely

( ) Sometimes

( ) Often

( ) Very Often

( ) I prefer not to answer

( ) Don't know

#### On a scale of 0 to 5, where 0 means "not very effective" and 5 means "very effective", how effective do you think this slogan or message is?

( ) 0 (Not Effective)

( ) 1

( ) 2

( ) 3

( ) 4

( ) 5 (Very Effective)

#### Greater than AIDS:

( ) Never

( ) Rarely

( ) Sometimes

( ) Often

( ) Very Often

( ) I prefer not to answer

( ) Don't know

#### On a scale of 0 to 5, where 0 means "not very effective" and 5 means "very effective", how effective do you think this slogan or message is?

( ) 0 (Not Effective)

( ) 1

( ) 2

( ) 3

( ) 4

( ) 5 (Very Effective)

#### Start Talking. Stop HIV.

( ) Never

( ) Rarely

( ) Sometimes

( ) Often

( ) Very Often

( ) I prefer not to answer

( ) Don't know

#### On a scale of 0 to 5, where 0 means "not very effective" and 5 means "very effective", how effective do you think this slogan or message is?

( ) 0 (Not Effective)

( ) 1

( ) 2

( ) 3

( ) 4

( ) 5 (Very Effective)

#### HIV Treatment Works:

( ) Never

( ) Rarely

( ) Sometimes

( ) Often

( ) Very Often

( ) I prefer not to answer

( ) Don't know

#### On a scale of 0 to 5, where 0 means "not very effective" and 5 means "very effective", how effective do you think this slogan or message is?

( ) 0 (Not Effective)

( ) 1

( ) 2

( ) 3

( ) 4

( ) 5 (Very Effective)

## Study Target Inquiries

#### As far as you know, did you participate in the “Sex is the Question” Survey between September 2016 and February 2017?

( ) Yes

( ) No

( ) I'm not sure

### For this national study, we are recruiting a large number of men like you. Can you tell us the name of social networking website or app where we could reach other men like you who might like to complete this survey?

_________________________________________________

## Future Contact

#### The PRISM Health team conducts many research projects at Emory University. Would you like to be contacted for potential participation in our future projects?

( ) Yes

( ) No

### Please provide the email address you would like for us to use to contact you for future studies.

_________________________________________________
